# Exercise training exerts beneficial effects on Alzheimer’s disease through multiple signaling pathways

**DOI:** 10.3389/fnagi.2025.1558078

**Published:** 2025-05-21

**Authors:** Jihe Kang, Mei Liu, Qiang Yang, Xiangji Dang, Qun Li, Ting Wang, Bin Qiu, Yibao Zhang, Xudong Guo, Xiaoling Li, Yan Liu

**Affiliations:** ^1^Department of Rehabilitation Medicine, Lanzhou University Second Hospital, Lanzhou, China; ^2^Department of Neurosurgery, Lanzhou University Second Hospital, Lanzhou, China; ^3^Department of Pharmaceutical, Lanzhou University Second Hospital, Lanzhou, China; ^4^Ultrasound Medical Center, Lanzhou University Second Hospital, Lanzhou, China; ^5^School of Pharmacy, Lanzhou University, Lanzhou, China

**Keywords:** exercise training, Alzheimer’s disease, signaling pathway, Aβ metabolism, tau pathology, PI3K/Akt signaling pathway, Wnt/β-catenin signaling pathway

## Abstract

Alzheimer’s disease (AD) is a neurodegenerative disease characterized by progressive memory loss and cognitive dysfunction that affects millions of people worldwide, placing a massive burden on families and economies. Exercise training can effectively reduce the prevalence of AD and alleviate its symptoms through the modulation of multiple signaling pathways involved in the pathophysiological process of AD, including the PI3K/Akt, Wnt/β-catenin, AMPK-related, MAPK, NF-κB, PINK1-PARKIN, JAK/STAT, and TREM2 signaling pathways. Different signaling pathways also crosstalk with each other through different targets to inhibit the formation of Amyloid β (Aβ) plaques, reduce the level of hyperphosphorylated tau protein, reduce apoptosis, relieve neuroinflammation, reduce autophagy dysfunction, and ultimately improve cognitive impairment in AD patients. This review summarizes the pathophysiological processes of AD affected by exercise training through different signaling pathways. We further provide a reference for the future development of new effective AD prevention and treatment targets to develop promising personalized, combined intervention strategies.

## 1 Introduction

Alzheimer’s disease (AD) is a neurodegenerative disease characterized by progressive memory loss and cognitive dysfunction. The World Report on Alzheimer’s Disease estimates that dementia affects approximately 50 million people worldwide, with a high incidence expected to triple by 2050 (Scheltens et al., 2021). Among the different forms of dementia, AD is the most common, accounting for an estimated 60–80% of dementia cases, exerting a significant care and economic burden on both societies and families (Better, 2023).

The primary pathological features of AD are the accumulation of extracellular Amyloid-β (Aβ) plaques and neurofibrillary tangles formed by hyperphosphorylation of microtubule-associated protein tau in neurons. In the ATN framework, AD is defined by the simultaneous deposition of two biomarkers, β-amyloid (A) and pathological tau (T), followed by neuronal damage or neurodegeneration (N) (Jack et al., 2018). Aβ accumulation can impair neuronal function by affecting inter-synaptic signaling, while tau tangles may block the transport of nutrients and molecules within neurons (Sato et al., 2018). Both may also be associated with mitochondrial abnormalities, neuroinflammation, lipid metabolism, iron metabolism, neuronal apoptosis, oxidative stress, etc. (Pahlavani, 2023). However, the specific mechanisms of action Aβ plaques and pathological tau deposition in the pathogenesis of AD remain unclear.

**Figure 1:**
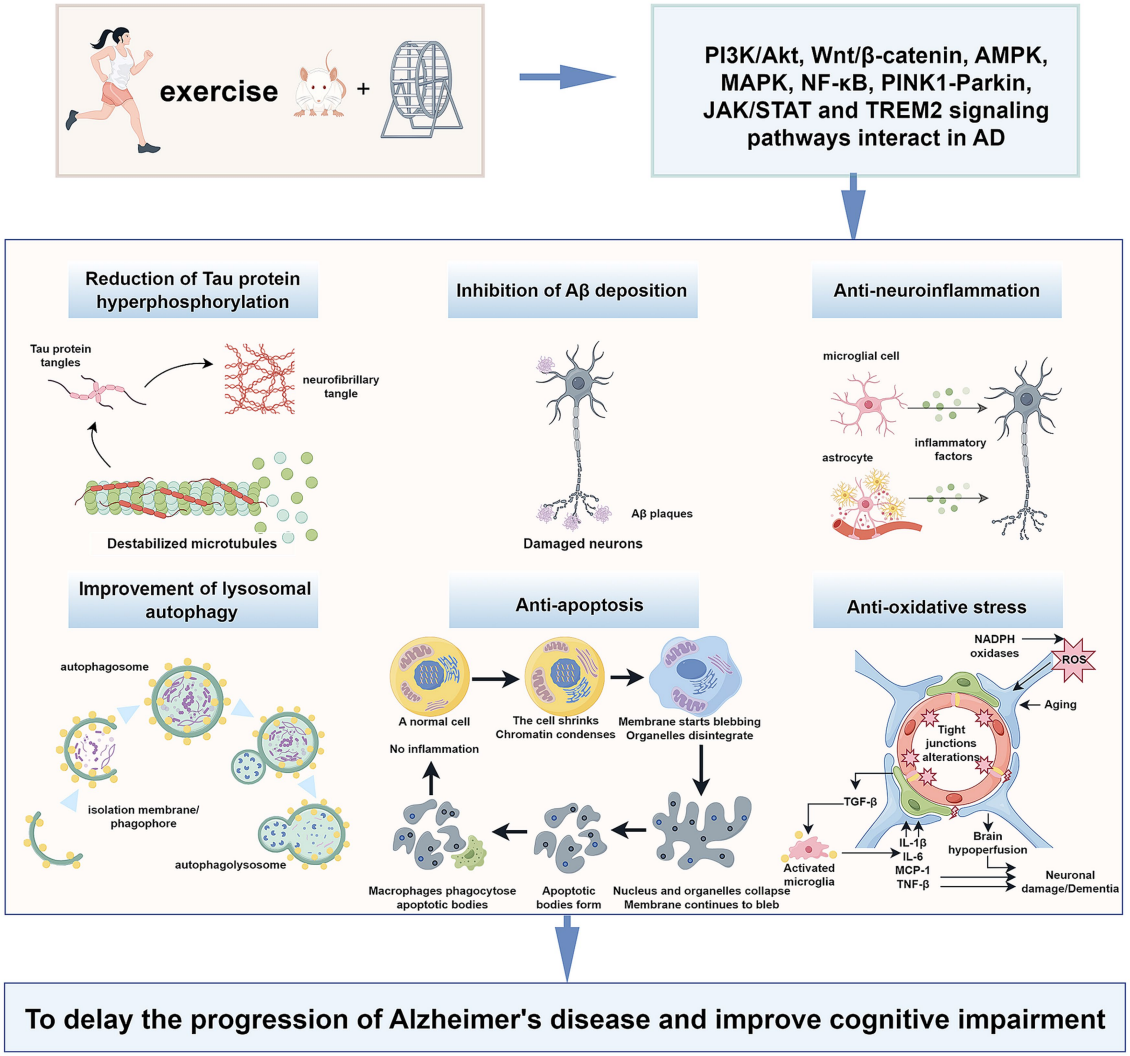
Exercise training exerts beneficial effects on Alzheimer’s disease through different signalling pathways.

Several prior meta-analyses and randomized controlled trials have found that moderate intensity and long-term continuous exercise training can effectively reduce the prevalence of AD and improve their cognitive status, physical performance, quality of life, and activities of daily living (Norton et al., 2014; López-Ortiz et al., 2021; de Sá Leitão et al., 2025). Indeed, research has identified 12 potentially modifiable risk factors for dementia identified during the processes of dementia prevention, intervention, and care. Adjustment of these AD-associated factors can prevent or delay the onset of AD in 40% of patients. Exercise training can reduce many risk factors, including diabetes, obesity, and hypertension, to better prevent and intervene in AD (Livingston et al., 2020). The benefits of exercise training in AD may stem from changes in the brain, including improvements in vascular function, brain glucose metabolism, and anti-inflammatory responses (Abdullahi et al., 2024). Studies have shown that exercise training activates a variety of signaling pathways involved in the pathophysiological process of AD, such as the phosphatidylinositol 3-kinase (PI3K) / protein kinase B (Akt), nuclear factor kappa B (NF-κB), Wnt/β-catenin, adenosine 5′-monophosphate-activated protein kinase (AMPK)-related, PTEN-induced kinase 1 (PINK1)-Parkin RBR E3 ubiquitin-protein ligase (PARKIN), and Triggering receptor expressed on myeloid cells 2 (TREM2) signaling pathways (Figure 1). These effects down-regulate the downstream factors of the above signaling pathways. Consequently, they inhibit Aβ plaque formation, reduce hyperphosphorylated Tau protein levels and apoptosis, alleviate neuroinflammation, mitigate autophagy dysfunction, and enhance lysosomal function (Table 1). We will explore the signaling pathways and molecules mediated by exercise that can serve as potential therapeutic targets for AD. Understanding the molecular pathways underlying the beneficial effects of exercise on AD will facilitate the promotion of exercise training in healthy populations and AD patients, as well as the development of new therapeutic targets and strategies for AD.

**Table 1:** Effects of different intensities of exercise training on AD animal models through different signaling pathways.

Experimental model	Exercise intervention	Tissue	Changes in detection indicators	Signaling pathways and mechanisms	Studies
Male APP/PS1 transgenic mice, age: 3 months	5 times of 45 min exercise per week, 12 weeks	Hippocampus	p-PI3k, p-Akt↑; Aβ peptide, Aβ plaque area↓; p-tau↓; HSP70, CHIP↑; BACE1↓; UCHL-1↑; Escape latency↓	Activation of PI3K/Akt pathway, promotion of clearance of β-amyloid and hyperphosphorylated tau by E3 ubiquitin ligase	Xu et al. (2022)
Tg-NSE/hPS2m transgenic mice, age: 24 months	5 times of 60 min exercise per week, 3 months	Cerebral cortex	p-PI3k, p-Akt↑; Bcl-2/Bax, HSP-70, BDNF↑; caspase-3, COX-2↓; Escape latency↓	Activation of PI3K/Akt pathway, reduction of neuronal apoptosis and cognitive improvement	Koo et al. (2013)
Male SD rats on a high-fat diet were fed on a high-fat diet for 8 weeks	5 times of exercise with progressively increasing training time per week, 4 weeks	Prefrontal cortex	p-PI3k, p-Akt↑; p-tau/tau↓; NMDAR, SYN↑; FOXO1, NF-κB, NLRP3↓	Inhibition of FOXO1/NF-κB/NLRP3 inflammatory signalling pathway and activation of PI3K/Akt/mTOR/4EBP2 pathway	Wang et al. (2019)
NSE/htau23 transgenic mice, age: 18 months	5 times of 20 min exercise per week, 12 weeks	Cerebral cortex	p-PI3k, p-Akt↑; p62, mTOR↓; LC3-B, Beclin-1, p-GSK3β/ t-GSK3β↓; hyperphosphorylated tau, escape latency↓	Activation of PI3K/Akt pathway, reduction of the activities of mTOR and GSK-3β, promotion of autophagy and reduction of tau hyperphosphorylation	Kang and Cho (2015)
Male diabetes-induced Sprague–Dawley rats, age: 7 weeks	5 times of 30 min exercise per week, 12 weeks	Hippocampus	p-PI3k, p-Akt↑; GSK-3β↓; the number of tau-positive cells↓; the latency of the avoidance task↓	Activation of PI3K/Akt pathway, reduction of GSK-3β activity, reduction of tau hyperphosphorylation	Kim et al. (2015)
AD mice (intraperitoneal injection of D-galactose and aluminum trichloride), age: 3 months	5 times of 20 min exercise per week, 8 weeks	Hippocampus	p-PI3k, p-Akt↑; GSK-3β↓; Bcl-2/Bax↑; The escape latency↓	Activation of PI3K/Akt/GSK-3β pathway, reduction of apoptosis, cognitive improvement	Peng et al. (2022)
Male SD rats on a high-fat diet, age: 28 weeks	5 times of 30 min exercise per week, 8 weeks	Cerebral cortex	p-PI3k, p-Akt↑; GSK-3β↓; hyperphosphorylated tau, escape latency↓	Activation of PI3K/Akt pathway, reduction of GSK-3β activity, reduction of tau hyperphosphorylation	Jeong and Kang (2018)
Male Sprague–Dawley rats, age: 5 weeks	5 times of 30 min exercise per week, 36 weeks	Hippocampus	AXIN1, Dkk-1, p-GSK-3α/β↓; LPR6, AXIN2, Bcl-2↑	Activation of Wnt pathway, anti-apoptosis	Bayod et al. (2014)
Naturally aged male rats, age: 21 months	3 times of 60 min exercise per week, 12 weeks	Hippocampus	p-β-catenin, active β-catenin↓; p-PI3K, p-AKT/AKT↑; Sirt1, SYN, PSD93, PSD95, p-GSK-3β (ser9) /GSK-3β, Bcl-2↑; DKK-1, AXIN1, Ac-p53/p53, p-CREB, BDNF, p-tau/tau, Bax↓; Escape latency, number of apoptotic neurons↓; Swimming speed, number of cross-platform↑	Activation of Wnt/β-catenin and PI3K/Akt pathways, reduction of GSK-3β activity, reduction of tau hyperphosphorylation, anti-apoptosis and cognitive improvement	Chen et al. (2020)
AD male rats (Aβ microinjected into the CA1 region of the hippocampus), age: adult (200–250 g)	5 times of exercise with progressively increasing training time per week, 4 weeks	Hippocampus	p-AMPK↑; PGC-1α, FNDC5, BDNF mRNA, PGC-1α, BDNF↑; travel distance, escape latency↓	Activation of AMPK and up-regulation of PGC-1α/FNDC5/BDNF pathway, improvements in spatial learning and memory	Azimi et al. (2018)
Male Sprague–Dawley rats (intraperitoneally injected with D-galactose), age: 3 months	5 times of exercise with progressively increasing training time per week, 12 weeks	Hippocampus	p-AMPK, SIRT1, PGC-1α, p-FOXO3a, SOD2↑; TNFα, p-NFκB, COX-2, iNOS, Fas ligand, FADD, activated caspase 8, Bad, cytochrome c, activated caspase 9, activated caspase 3↓	Activation of AMPK/SIRT-1/PGC-1α/FOXO3a and IGFI-R/PI3K/Akt pathways, inhibition of hippocampal inflammation and apoptosis	Lin et al. (2020)
APP/PS1 transgenic mice, age: 6 months	5 times of 37 min exercise per week, 9 weeks	Hippocampus	SIRT1, PGC-1α, PPARγ↑; BACE1, IL-1β, IL-6, TNF-α, ROS, H2O2, MDA↓; escape latency, neuronal damage↓; number of cross-platform↑	Activation of SIRT1/PGC-1α pathway, cognitive improvement, reduction of oxidative stress, anti-inflammation, reduction of neuronal damage and Aβ plaques	Shi et al. (2023)
NSE/APPsw transgenic AD mice, age: 12 months	5 times of exercise with progressively increasing training time per week, 12 weeks	Cerebral cortex	SIRT1, PGC-1α, RARβ, ADAM-10↑; BACE-1, C-99, Aβ, ROCK-1, cleaved caspase-3, TUNEL-positive cells, Escape latency↓; m-cyto c/c-cyto c↑	Activation of SIRT-1/PGC-1α pathway, activation of non-amyloid-producing pathway and inhibition of amyloid-producing pathway	Koo et al. (2017)
Male diabetes-induced Wistar rats (high-fat diet and STZ injection for 2 months), age: 7 weeks	5 times of 10 min exercise with training intensity gradually increased per week, 8 weeks	Serum and hippocampal tissue	p-AMPK↑; DeP-GSK3β, p-tau↓; Blood glucose, insulin sensitivity index↓; Adiponectin, insulin, InsRB, APNR1, APNR2↑	Activation of AMPK pathway, reduction of dephosphorylated GSK3β and tau hyperphosphorylation	Khoramipour et al. (2023)
AD transgenic mice and context-matched WT mice, age: 5 months	6 times of 40 min exercise per week, 5 months	Prefrontal cortex and hippocampus	p-AMPK, p-ULK1 (Ser 555), ACSS2, total TFEB, nuclear TFEB↑; Aβ, βCTF, P-tau↓; Atg5-Atg12, Rab7, Rab9↑; p62, LC3II, LAMP1↓	Activation of AMPK pathway, enhanced ACSS2 interaction with TFEB and enhanced lysosomal function	Wang et al. (2022b)
Male APP/PS1 double transgenic mice, age: 8 weeks/male wild-type mice, age: 6 weeks	5 times of 45 min exercise per week, 3 months	Hemibrains	p-AMPK, PLC/Kcnip3/Mid1/PP2A↑; Adiponectin, LAMP1, Atp6v1h, APNR1, Aβ, Beclin1, LC3-II/I↑; Aβ, Bax, mTOR, P62, Escape latency, dendritic spine density↓	Activation of AdipoR1/AMPK/TFEB pathway, enhancement of lysosomes and alleviation of aberrant autophagy, remission of Aβ deposition and its associated AD-like abnormalities	Jian et al. (2022)
Male diabetes-induced SD rats (STZ injection and high-fat diet for 4 months), age: 8 weeks	7 times of 60 min exercise per week, 8 weeks	Cortex and hippocampus	PI3k, p-Akt, p-AMPK, p-mTOR↑; p-GSK-3β↓; Aβ, BACE1, PHF10↓; GLUT4↑; escape latency, neuronal damage↓; number of cross-platform↑	Activation of PI3K/AKT//ERK and AMPK/mTOR signalling pathways, cognitive improvement, enhanced GLUT4 transport	Zhang et al. (2022c)
PS2 mutant mice, age: 24 months	5 times of 60 min exercise per week, 12 weeks	Cortex and hippocampus	p-JNK 54, p-JNK 46, p-p38MAPK, p-PERK, p-eIF2α↓; Bcl-2↑; Aβ-42, Aβ plaque, BACE1, C-99, GRP78/BiP, PDI↓; CHOP, caspase-12, caspase-3, TNFα, IL-1α, Bax, TUNEL positive cell number↓; Escape latency, escape distance↓;	Inhibition of UPR and JNK-p38 MAPK signalling pathway, reduction of neuronal cell death and inflammation caused by Aβ-induced ER stress	Kang et al. (2013)
AD male rats (Aβ injected into the lateral ventricle), age: 40 weeks	7 times of 30 min exercise per week, 4 weeks	Hippocampus	NF-κB, p-IκB, p-ERK, p-JNK, p-p38↓; TNF-α, IL-1β, Bax↓; Bcl-2↑; number of TUNEL-positive cells, escape latency↓	Inhibition of NF-κB and MAPK signalling pathways, reduction of inflammation and apoptosis, cognitive improvement	Kim et al. (2021)
AD male mice (Aβ injection into bilateral hippocampus), age: 8 weeks	1 times of 30 min exercise per week, 1 weeks	Hippocampus	p-ERK, p-JNK, p-p38↓; TNF-α, IL-1β↓; Neurogenesis↑; hippocampal astrocytes, escape latency↓	Inhibition of MAPK signalling pathway, regulation of hippocampal neurogenesis and inflammatory responses	Sun et al. (2018)
AD 5xFAD mouse model, age: 6 weeks	Free exercise for 6 months	Cortex	IL-6R mRNA, Jak1 mRNA, STAT3 mRNA↓; PTPe mRNA, TfR↑; Hepcidin, Aβ, HO-1, ferritin, TfR mRNA, DMT1 mRNA, ceruloplasmin gene↓	Reduction of hepcidin in the brain via the IL-6/STAT3/JAK1 pathway	Belaya et al. (2021)
Male APP/PS1 transgenic mice, age: 3 months	5 times of 45 min exercise per week, 12 weeks	Hippocampus	PINK1↓; Parkin↑; P62, body weight, Aβ peptide, Aβ plaque area ↓; LC3II, SYN, GAP43, PGC-1α, TFAM↑; damaged mitochondria, escape latency↓; number of platform crossings↑	Through PINK1/Parkin pathway, mitochondrial dysfunction was reversed and learning ability was improved	Zhao et al. (2020)
Male APP/PS1 transgenic mice, age: 3 months,	5 times of 45 min exercise per week, 12 weeks	Hippocampus	PINK1, Ace-FOXO1a (Lys294), Ace-FOXO3a (Lys271) ↓; Parkin, SIRT1↑; P62, Aβ40, Aβ42, Aβ plaque area↓; LC3II/I, ATP, complex I, Complex IV↑; number of platform crossings↑	Activation of SIRT1-FOXO3-BNIP3 axis initiates PINK1-Parkin-mediated mitophagy	Zhao et al. (2023)
Tg-NSE/htau23 mice, age: 16 months	5 times of 60 min exercise per week, 12 weeks	Brain tissue	p65, p-p38, p-ERK1/2, TNF-α, IL-1β, IL-6, iNOS, COX-2, GFAP + and MAC-1 + cells in hippocampus↓	It exerted anti-inflammatory effects and attenuated the activation of microglia and astrocytes through the MAPK-dependent NF-κB pathway	Leem et al. (2011)
Swiss Albino mice (Aβ injected into the lateral ventricle), age: 3 months	5 times of 60 min exercise with training intensity gradually increased per week, 8 weeks	Prefrontal cortex and hippocampus	NF-κB, IDO, KYN/TRP↓; BDNF, GDNF, NGF, NT-3, citrate synthase ↑; IL-6, IL-4↓; Identification index, open arms time ↑; Immobile time↓	Inhibition of NF-κB /IDO activation, anti-inflammation and neuroprotection	Souza et al. (2017)
AD male rats (Aβ injected into the lateral ventricle), age: 2 months	5 times of 60 min exercise per week, 4 weeks	Hippocampus	TREM2, HSP60↑; Nf-κB, Aβ, IL-1β, TNF-α, iNOS, CD16↓; IL-4, IL-10, Arg-1, CD206↑	Exercise training inhibits microglia activation and inflammation in a HSP60/TREM2/ DAP12-dependent manner	Zhang et al. (2022a)

## 2 Exercise training affects different signaling pathways in AD

### 2.1 PI3K/Akt signaling pathway

**Figure 2:**
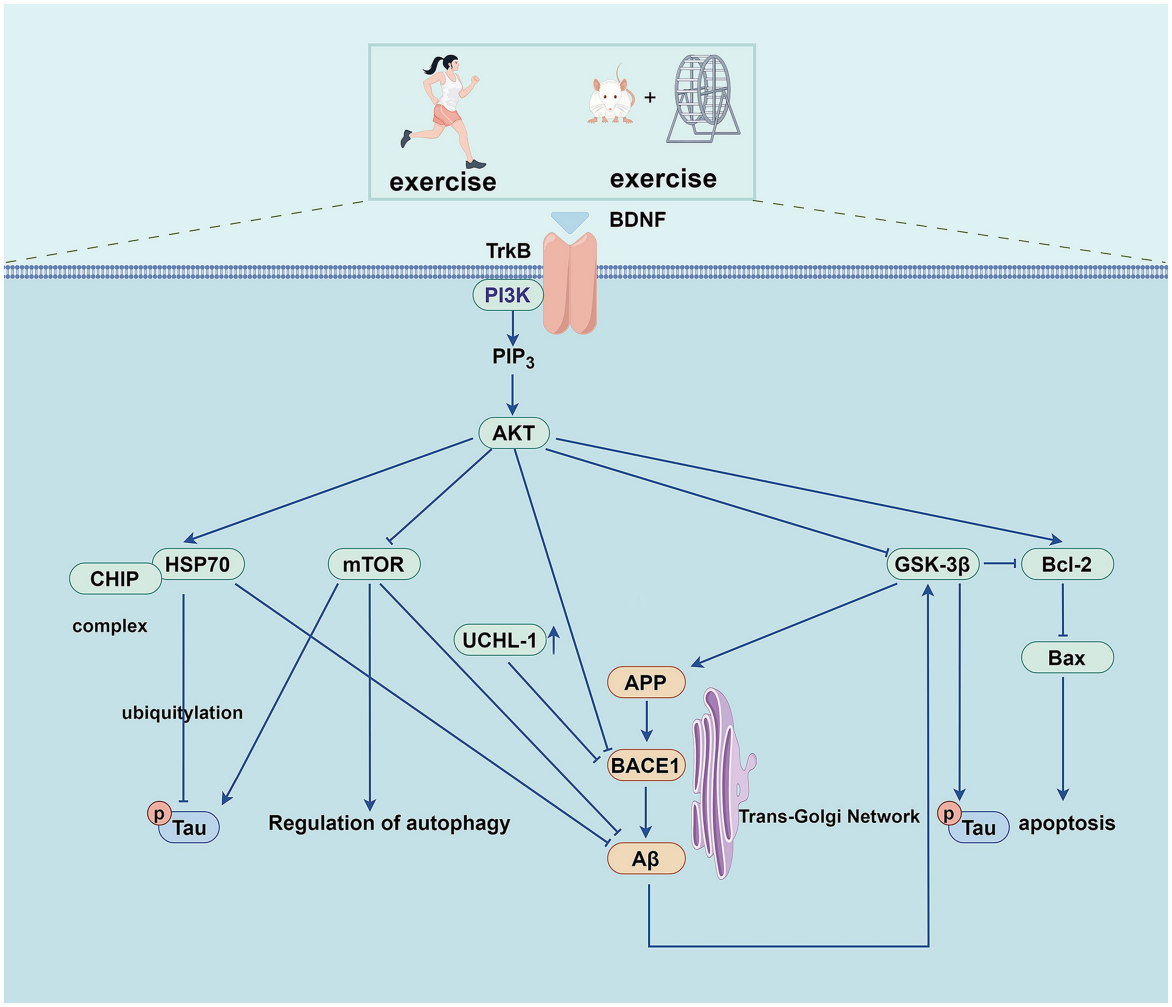
Exercise training exerts beneficial effects on AD through PI3K/AKT pathway.

The class I isoform of PI3K is a heterodimeric structure comprising a regulatory p85 subunit and a catalytic p110 subunit. The catalytic subunit of p110 includes the p110α, p110β, p110γ, and p110δ domains (Vanhaesebroeck et al., 2010). Upon receiving an upstream signal, the p85 subunit is recruited to the plasma membrane. It binds to the p110 subunit, phosphorylating phosphatidylinositol-(4,5) diphosphate to phosphatidylinositol (3,4,5)-triphosphate, which subsequently binds Akt and transfers it from the cytoplasm to the cell membrane for activation. Furthermore, it phosphorylates a variety of downstream effector proteins (Wymann et al., 2003), including 70-kDa heat shock protein (Hsp70), the mammalian target of rapamycin (mTOR), and glycogen synthase kinase-3β (GSK-3β), among others (Figure 2). The PI3K/Akt signaling pathway is involved in cell survival, autophagy, apoptosis (Kumari et al., 2023), Aβ deposition, cell senescence, neurofibrillary tangle formation (Kumar and Bansal, 2022), mitochondrial dysfunction, and glucose metabolism (Wang et al., 2023a).

The expression of HSP70 is transcriptionally regulated by heat shock transcription factor 1 (HSF1), and the PI3K/Akt pathway regulates the translational regulation of HSF1 (Zhou et al., 2004). At the same time, some studies have found that HSP70 can also affect the phosphorylation of the PI3K/Akt/mTOR pathway (Dokladny et al., 2013). HSP70 is a molecular chaperone involved in the entire life cycle of various cellular proteins, from synthesis to degradation, which plays a vital role in multiple diseases. In AD, HSP70 can bind to amyloid precursor protein (APP) and interfere with its secretion, thereby reducing the formation of Aβ. Further, it can promote the degradation of tau and Aβ oligomers through the proteasome system (Maiti et al., 2014). Studies have demonstrated that the PI3K/Akt pathway regulates endogenous HSF1 and HSP70 expression, suppresses Aβ aggregation, and inhibits neuronal apoptosis. Through these mechanisms, it exerts a neuroprotective effect against AD (Ren et al., 2020). Treadmill exercise has been shown to activate the PI3K/Akt signaling pathway and upregulate HSP70 protein expression in the hippocampus of AD mice, ultimately reducing Aβ deposition and tau phosphorylation (Um et al., 2011). Treadmill exercise can also increase the expression of brain-derived neurotrophic factor (BDNF) in the cortex of mice, as well as increase the expression of HSP70 by activating the PI3K/Akt signaling pathway to improve the cognitive dysfunction of mice induced by Aβ. In addition, treadmill exercise exerts neuroprotective effects by inhibiting neuronal apoptosis (Koo et al., 2013).

The ubiquitin-proteasome system (UPS) is a selective proteolytic system that degrades misfolded or aggregated proteins, and its dysfunction is closely related to neurodegenerative diseases (Liang et al., 2023). The PI3K/Akt pathway inhibits transcription of E3 ubiquitin ligases that regulate UPS-mediated protein degradation (Yoshida and Delafontaine, 2020). The Carboxyl terminus of the HSP70 interacting protein (CHIP) acts as an E3 ubiquitin ligase in the UPS; it can form a complex with HSP70 and present pathological tau protein to the proteasome for degradation, which is closely related to the occurrence and development of AD (Petrucelli et al., 2004). One study (Xu et al., 2022) found that the messenger ribonucleic acid (mRNA) levels of HSF1 and HSP70 and the protein levels of HSP70 and CHIP were decreased in the hippocampus of APP/presenilin-1 (PS1) transgenic mice. Further research has shown that treadmill training activates the PI3K/Akt pathway and up-regulates the protein expression of HSP70 in AD mice’s hippocampus while increasing CHIP’s protein expression. This effect may be attributed to the upregulation of HSP70 and compensatory CHIP protein activity. These changes reduce soluble phosphorylated tau deposition and neuronal fibrillary tangle formation, thereby enhancing cognitive function in APP/PS1 transgenic mice.

Aβ is mainly produced by the proteolysis of APP through β-secretase (BACE) (including BACE1 and BACE2) and *γ*-secretase. Activation of the PI3K/Akt signaling pathway reduces the levels of BACE1 and γ-secretase, thereby reducing the formation of Aβ and further alleviating AD (He et al., 2016). As A deubiquitinating enzyme in the UPS, ubiquitin carboxyl-terminal hydrolase L1 (UCHL-1) is involved in the degradation of APP and BACE1. Still, the expression of UCHL-1 is decreased in the early stage of AD, which eventually leads to the increase of Aβ expression (Zhang et al., 2012; Choi et al., 2004). The mRNA and protein levels of BACE1 in the hippocampus of APP/PS1 Tg mice are significantly increased, while the expression of UCHL-1 protein is significantly decreased in the pathological state of AD. Studies have demonstrated that treadmill exercise activates the PI3K/Akt signaling pathway in the hippocampus of APP/PS1 mice. This activation downregulates BACE1 expression while upregulating UCHL-1 levels, ultimately enhancing cognitive function in these transgenic mice (Xu et al., 2022).

As a downstream effector of the PI3K/Akt signaling pathway, mTOR is over-activated in the early stages of AD. mTOR is a serine/threonine kinase that forms the core of two multiprotein complexes, mTOR complex (mTORC)1 and mTORC2. In general, mTORC1 is involved in cell growth, glucose transport, autophagy, and protein synthesis, whereas mTORC2 is involved in cytoskeletal remodeling, electrolyte homeostasis, cell survival, autophagy etc. (Querfurth and Lee, 2021). mTOR signaling is involved in multiple processes in AD pathophysiology, including the formation and deposition of Aβ, tau hyperphosphorylation, neuroinflammation, autophagy, apoptosis, synaptic plasticity, vascular dysfunction, etc. (Davoody et al., 2024). The mTOR overactivation increases the activities of β-secretase and *γ*-secretase, negatively regulating autophagy, leading to the increased generation and deposition of Aβ (Perluigi et al., 2021; Caccamo et al., 2013). Research indicates that treadmill exercise activates the PI3K/Akt pathway in the cerebral cortex of NSE/htau23 transgenic mice. This activation modulates mTOR expression to suppress abnormal autophagy activity, ultimately reducing Aβ deposition and tau hyperphosphorylation in the same brain region. Finally, it improves the cognitive abilities of the mice (Kang and Cho, 2015). Another study showed that aerobic exercise activates the PI3K/Akt signaling pathway in the prefrontal cortex of rats, which regulates the protein expression of mTOR and reduces the hyperphosphorylation of tau (Wang et al., 2019).

GSK-3 is a serine/threonine kinase widely expressed in cells. The GSK-3β form is highly expressed in the brain and plays a vital role in neuronal survival, neurogenesis, and synaptic plasticity. Dysregulation of GSK-3β is associated with various diseases, including diabetes, obesity, and neurodegenerative diseases (Sayas and Ávila, 2021). As one of the significant kinases that phosphorylate tau, GSK-3β plays an intermediate role between Aβ and tau in the pathophysiological process of AD. When activated by Aβ, GSK-3β further phosphorylates the tau protein (Phiel et al., 2003). The hyperphosphorylated tau protein dissociates from microtubules, followed by microtubule destabilization and tau oligomerization, eventually forming neurofibrillary tangles in the cell.

On the other hand, GSK-3β regulates APP metabolism and Aβ production and promotes Aβ-induced neuronal death (Cheng et al., 2024). GSK-3β can further aggravate AD pathology by triggering inflammatory and apoptotic pathways (Kumari et al., 2023). Studies have shown that treadmill exercise reduces tau hyperphosphorylation by increasing PI3K/Akt phosphorylation and decreasing GSK-3β activity in the cerebral cortex of NSE/htau23 transgenic mice (Kang and Cho, 2015). In the diabetes-induced rat model (Kim et al., 2015) and the high-fat diet Sprague Dawley (SD) rat model (Jeong and Kang, 2018), aerobic exercise also reduced the activity of GSK-3β. It inhibited the hyperphosphorylation of tau protein by activating the PI3K/Akt pathway. Further, it improved the cognitive ability of rats. The gradual aggregation of neurofibrillary tangles leads to neuronal apoptosis. Aerobic exercise can reduce hippocampal cell apoptosis in AD mice by regulating the PI3K/Akt/GSK-3β pathway, increasing the expression of B-cell lymphoma-2 (Bcl-2) protein, and inhibiting the expression of Bcl-2-associated X protein (Bax). Consequently, it improves cognitive function and enhances learning and memory (Peng et al., 2022).

### 2.2 Wnt/β-catenin pathway

The Wnt/β-catenin pathway is involved in multiple processes in the pathophysiology of AD (Folke et al., 2019). The Wnt signaling pathway includes both the noncanonical and canonical pathways. The canonical Wnt pathway, also known as the Wnt/β-catenin pathway, involves the nuclear translocation of β-catenin and activation of target genes by related transcription factors. The upregulated genes are involved in cell proliferation, survival, and differentiation (Clevers, 2006). Without Wnt signaling, β-catenin is degraded by protein complexes, including AXIN, adenomatous polyposis coli, GSK-3β, etc. GSK-3β is inhibited when Wnt/β-catenin is activated. GSK-3β cannot phosphorylate β-catenin due to its increased activity and translocation to the nucleus, activating the transcription of Wnt target genes (Law and Zheng, 2022).

**Figure 3:**
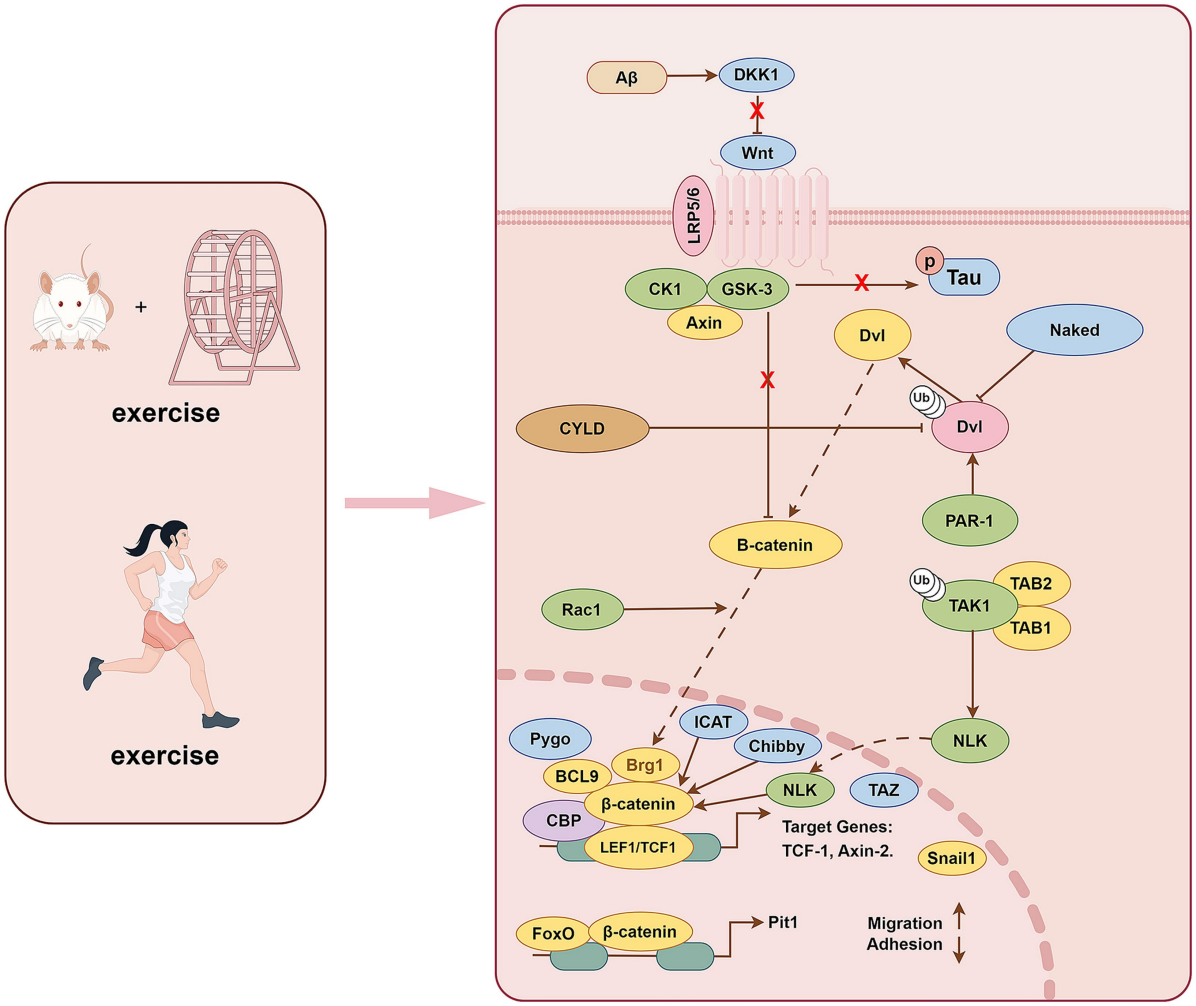
Exercise training exerts beneficial effects on AD through Wnt/β-catenin pathway.

Low-density lipoprotein receptor-related protein 6 (LRP6) is a key molecule in the cell membrane of the Wnt/β-catenin pathway and is down-regulated in the AD brain. Genetic variants in LRP6 lead to progressive synaptic dysfunction that manifests with aging, further revealing the relationship between aging and AD (Jones et al., 2023). LRP6-mediated Wnt/β-catenin signaling defects are essential in regulating synaptic function, blood–brain barrier function, and amyloid protein accumulation in AD (Liu et al., 2014; Wang et al., 2022a). The activation of noncanonical and canonical Wnt signaling alternatively increases and decreases Aβ production. Aβ induces the production of Wnt inhibitor Dickkopf-1 (Dkk1) and promotes GSK-3β activity. While shifting the balance from Wnt/β-catenin transduction to a noncanonical Wnt signaling (Elliott et al., 2018), GSK3β is activated in AD brains. It promotes the degradation of β-catenin by phosphorylating it, further contributing to the inactivation of the Wnt classical pathway. In summary, activation of the Wnt/β-catenin signaling pathway rescues Aβ-induced neurodegeneration and corresponding dysfunction (Liu et al., 2022a) (Figure 3).

Studies have shown that sedentary behavior leads to a high expression of the Wnt inhibitor Dkk1 in the rat hippocampus, whereas exercise reduces Dkk1 expression (Bayod et al., 2014). Sedentary behavior decreased the total protein level of LRP6 (Bayod et al., 2014), which may be related to the Dkk1-induced internalization of the LRP6 receptor (Yamamoto et al., 2008). The levels of AXIN1, a component of the destruction complex, and GSK-3α/β activation were shown to be reduced in the hippocampus of exercise-trained rats, but the different phosphorylated forms and total β-catenin protein levels did not change significantly (Bayod et al., 2014). It has also been found that GSK-3 colocates with Dkk-1 and phosphorylated tau in AD mice, and β-catenin nuclear translocation downstream of GSK-3 is significantly reduced, indicating that Wnt signaling pathway is functionally impaired (Rosi et al., 2010). In addition, exercise training increased the expression of AXIN2, a direct target of the Wnt pathway, which is mediated by T-cell factor/lymphoid enhancer factor factors and the anti-apoptotic protein Bcl-2. AXIN2 further regulates the duration/intensity of Wnt signaling through a negative feedback loop (Jho et al., 2002). As such, exercise training can promote the activation of the Wnt/β-catenin signaling pathway and inhibit the expression and activity of GSK-3β in the rat hippocampus, thereby reducing neuronal apoptosis (Bayod et al., 2014). The Wnt signaling pathway regulates neuronal survival and synaptic plasticity, and failure of this signaling leads to brain aging and cognitive dysfunction. In aging rats, the expression of the Wnt inhibitor Dkk1, GSK-3β activation, and hyperphosphorylated tau significantly increased (Chen et al., 2020).

Further, p-β-cateninSer33, 37, Thr41 is highly expressed and promotes neuronal degradation. At the same time, the expression of the apoptosis-related protein Bax is increased, and that of the anti-apoptosis-related protein Bcl-2 is decreased. However, treadmill exercise reversed these changes in the hippocampi of aged rats. Simultaneously, treadmill exercise activated the down-regulated PI3K/Akt AD’s physiological and pathological process and Wnt/β-catenin signaling pathway, alleviated synaptic toxicity and neuronal apoptosis, and improved cognitive dysfunction in aging rats (Chen et al., 2020). Studies have also identified a crosstalk pattern between Wnt/β-catenin and other signaling pathways, such as NF-κB and Notch, showing that they work together to regulate and enhance the synaptic plasticity of neurons during exercise (Methi et al., 2024). The activation and inactivation of various components of the exertion-related Wnt/β-catenin pathway are widely involved in AD’s physiological and pathological process. It is further revealed that the exercise-related Wnt/β-catenin pathway has practical value in clinical practice, and further studies on this pathway and its crosstalk with other signaling pathways are needed.

### 2.3 AMPK signaling pathway

**Figure 4:**
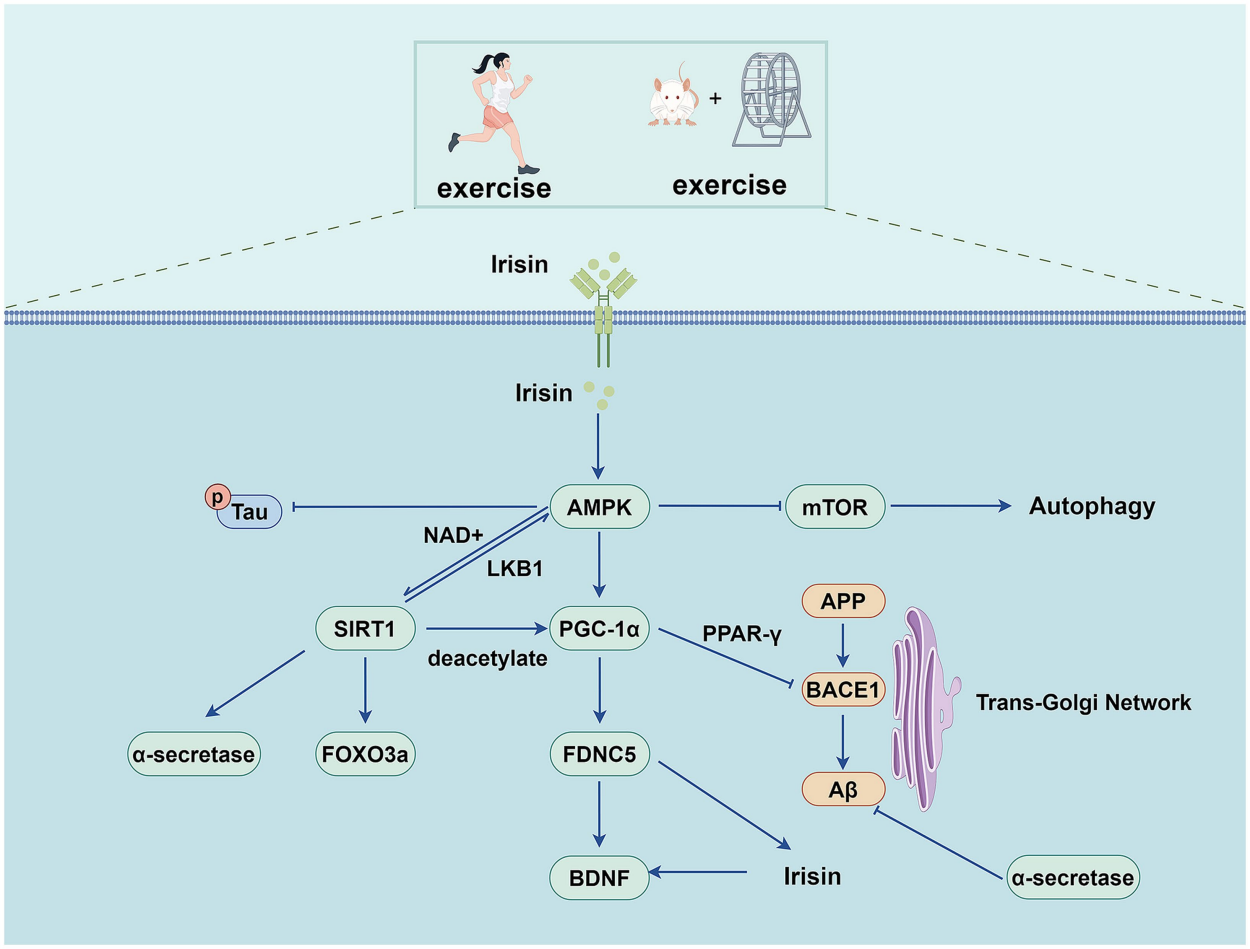
Exercise training exerts beneficial effects on AD through AMPK pathway.

In AD, AMPK is abnormally activated in both tangled preganglionic neurons and neurons containing tangles (Vingtdeux et al., 2011b), but it has also been reported that both resveratrol and small molecules can promote the autophagy-dependent degradation of Aβ peptide by activating AMPK (Vingtdeux et al., 2011a), this paradoxical result may also be related to the application of the highly nonspecific kinase inhibitor compound C in the experiments (Dasgupta and Seibel, 2018). The inhibition of AMPK ameliorates synaptic plasticity disruption caused by exogenous β-amyloid exposure or APP/PS1 transgenic mice (Ma et al., 2014). The pros and cons of AMPK activation and inhibition in AD development remain controversial. Still, they may be related to the type of nerve cells, the site and nature of the injury, and the intensity and duration of the AMPK activation (Muraleedharan and Dasgupta, 2022). The regulation of AMPK at different stages and in various stages of AD requires further investigation. Herein, we discuss the role and mechanism of exercise training in regulating AMPK expression in AD (Figure 4).

AMPK is a highly conserved serine/threonine kinase that forms as a heterotrimer containing catalytic *α* subunits and regulatory β and *γ* subunits (Herzig and Shaw, 2018). AMP binds to the γ subunit and constitutively activates the complex, making it more susceptible to phosphorylation at Thr 172 and phosphorylation by upstream liver kinase B1 in the activation loop of the α subunit. Metabolic hormones such as adiponectin and leptin stimulate changes in intracellular calcium levels, resulting in the direct phosphorylation of AMPK at Thr172 by calcium/calreticulin-dependent protein kinase 2 (Trefts and Shaw, 2021). Activated AMPK regulates the metabolic and cellular adaptive processes by phosphorylating various downstream effector proteins. AMPK signaling is essential for both the benefits of exercise training and physical health, while AMPK signaling activation enhances physical health primarily through mitochondrial dynamics (Campos et al., 2023).

Exercise training can activate the AMPK signaling pathway in various tissues and organs (including the skeletal muscle, brain, heart, and adipose tissue), thereby regulating glucose, lipid, and protein metabolism and processes such as autophagy and mitochondrial homeostasis (Spaulding and Yan, 2022). Some studies have also found that exercise training can alter the microbiota and reduce AMPK phosphorylation in the livers of APP/PS1 transgenic mice (Téglás et al., 2020). Further studies are required to clarify the relationship between gut microbiota, exercise training, and AD.

AMPK also enhances sirtuin1 (SIRT-1) activity by increasing the cellular concentration of nicotinamide adenine dinucleotide, a cofactor of the deacetylase SIRT-1, thereby promoting the deacetylation of downstream SIRT-1 targets and regulating their activity. These targets include peroxisome proliferator-activated receptor-γ coactivator-1α (PGC-1α) and the forkhead transcription factors forkhead box O (FOXO)1 and FOXO3a (Cantó et al., 2009). PGC-1α is a widely expressed transcriptional regulator regulating the expression of genes involved in mitochondrial biogenesis, regulating mitochondrial function, energy metabolism, oxidative stress, and neuroinflammation (Yang et al., 2023). Treadmill exercise induced increased expression of SIRT-1 and PGC-1α, which inhibited Aβ production by inhibiting BACE1 expression and activating the non-amyloid pathway. It alleviates pathological damage in AD and improves cognition by reducing neuroinflammation, apoptosis, and oxidative stress (Koo et al., 2017; Shi et al., 2023). Another study found that swimming exercise inhibited inflammation and apoptosis in the aging-induced hippocampus of male rats by activating the AMPK/SIRT-1/PGC-1α/FOXO3a and insulin-like growth factor (IGF)1/PI3K/Akt signaling pathways (Lin et al., 2020). Fibronectin type III domain containing 5 (FNDC5) is a PGC-1α-dependent transmembrane precursor protein highly expressed in the skeletal muscle and brain during exercise. FNDC5 is cleaved to produce irisin, a soluble polypeptide. This, in turn, promotes BDNF expression in the hippocampus (Liu et al., 2022b; Belviranlı and Okudan, 2018). Irisin is a myofactor that has neuroprotective properties and promotes neuronal survival. Irisin can also enhance cognitive function and synaptic plasticity, providing potential therapeutic utility for AD (Ratne et al., 2025). FNDC5/irisin levels are reduced in the cortex and cerebrospinal fluid of human patients and mouse models of AD. Increased brain or peripheral FNDC5 and irisin levels can rescue synaptic and memory dysfunction in mice with AD. Blockade of brain or peripheral FNDC5/irisin attenuated the beneficial effects of physical exercise on synaptic and memory dysfunctions in AD mice (Lourenco et al., 2019). Pretreatment with CoQ10 and HIIT improved the Aβ-induced reduction in BDNF levels probably through the FNDC5/irisin pathway and preventing Aβ plaque formation (Puoyan-Majd et al., 2025). Studies have shown that exercise training up-regulates the AMPK/PGC-1α/FNDC5/BDNF pathway in the hippocampus of AD rats while ameliorating Aβ-induced learning and memory impairment (Azimi et al., 2018).

Adiponectin (ADPN) is a hormone derived from adipocytes involved in various metabolic pathways. Accumulating evidence suggests that adiponectin exerts neuroprotective effects. Indeed, studies have found that inhibiting adiponectin receptor 1 (AdipoR1) leads to spatial learning and memory impairment, and AD-like pathological manifestations, including insulin signaling dysfunction, abnormal protein accumulation, and neuroinflammation (Kim et al., 2017). ADPN further activates AMPK through adiponectin receptors, while AMPK phosphorylation induces MPK-mediated nuclear translocation of acetyl-CoA synthetase 2 (ACSS2) (Li et al., 2017). ACSS2 subsequently binds to transcription factor EB (TFEB) in the nucleus and enhances TFEB-regulated genes. As a significant transcriptional regulator, TFEB has been implicated in pathophysiological processes, including mitochondrial and energy metabolism, lysosomal biogenesis, autophagy, oxidative stress, and inflammation (Abokyi et al., 2023). In diabetic rats, the expressions of insulin, ADPN, and their corresponding receptors were found to be decreased, and the expression of AMPK in the hippocampus was reduced. At the same time, the levels of dephosphorylated GSK3-β and tau were increased. Exercise training increases insulin and ADPN content, activates AMPK, and decreases dephosphorylated GSK3β and tau hyperphosphorylation (Khoramipour et al., 2023). Exercise training promotes the nuclear translocation of TFEB, activates AMPK, and promotes the nuclear translocation of ACSS2 and the interaction between ACSS2 and TFEB in AD mice, thereby regulating the transcription of genes involved in lysosomal biogenesis to promote lysosomal biosynthesis. By activating lysosomal enzyme maturation (mediated by vesicular transport proteins) and boosting autophagy, this process restores impaired autophagic flux in AD mouse brains. These coordinated effects improve Aβ catabolism, preserve proteostasis, and decelerate Alzheimer’s pathogenesis (Wang et al., 2022b).

Exercise training further reduces Aβ deposition and AD-like lesions in AD mice by activating the AdipoR1/AMPK/TFEB signaling pathway, enhancing lysosomal function, and ameliorating abnormal autophagy. Among these processes, the AdipoR1/ phospholipase C (PLC)/ protein phosphatase 2A (PP2A) signaling pathway may play an important role in exercise training, promoting TFEB nuclear translocation and enhancing the autophagy-lysosomal path in the brain cells of AD mice (Jian et al., 2022). AMPK also regulates mTOR signaling via two different pathways. Studies have found that exercise training can reverse autophagy defects by upregulating the AMPK/mTOR signaling pathway (Wu et al., 2024). Exercise training has also been shown to improve cognitive impairment and enhance glucose transporter type 4 (GLUT4) transport in rats with diabetic encephalopathy by modulating the reduction of growth factor receptor binding protein-10 in hippocampal and cortical tissues via the AMPK/mTOR signaling pathway (Zhang et al., 2022c). Exercise-induced AMPK activation can play a vital role in the pathophysiology of AD by promoting relevant metabolic and cellular adaptations and further improving energy homeostasis. However, the exact mechanisms by which exercise training-induced AMPK affects AD may be multifaceted and require further investigation.

### 2.4 MAPK signaling pathway

Mitogen-activated protein kinase (MAPK) are serine and threonine kinases capable of translating extracellular stimuli into various cellular responses. Different stimuli activate different MAPK pathways. Extracellular signal-regulated kinase (ERK), c-Jun N-terminal kinase (JNK), and p38 (*α*, β, *γ*, and *δ*) families have been widely studied (Cargnello and Roux, 2011). The JNK and p38 family, also known as stress-related protein kinases, are also involved in neuroinflammation, β-amyloid deposition, tau phosphorylation, synaptic plasticity, and other processes associated with AD (Munoz and Ammit, 2010; Hasegawa et al., 2018). It plays crucial roles in exercise-induced skeletal muscle adaptation, reactive oxygen species (ROS) production, and mitochondrial biogenesis (Reisman et al., 2024).

p38MAPK is associated with tau hyperphosphorylation. One *in vitro* study found that the p38MAPK pathway was activated, which mediated tau hyperphosphorylation and increased apoptosis in neurons exposed to glucose deprivation stress (Lauretti and Praticò, 2015). *In vivo* experiments using transgenic h-tau animals as a model have confirmed that abnormal tau hyperphosphorylation and aggregation are mediated by glucose hypometabolism and activation of the p38 MAPK pathway (Lee and Kim, 2017; Lauretti et al., 2017). It was also found that Aβ induced the hyperphosphorylation of p38 and JNK and inhibited the phosphorylation of ERK in the hippocampus of AD mice, which could be alleviated by exercise training. Exercise training rescued Aβ-induced cognitive dysfunction in AD mice by increasing adult hippocampal neurogenesis and attenuating hippocampal immune responses (Sun et al., 2018).

In vivo experiments have also found that the abnormal aggregation of Aβ can cause chronic endoplasmic reticulum stress, consequently causing dysregulation of the unfolded protein response (UPR) signaling pathway and cellular stress response, further activating MAPK kinases such as JNK and p38 and promoting AD-mediated neuronal apoptosis and neuroinflammation. However, exercise training suppresses two key pathological drivers: activation of the UPR signaling pathway and JNK-p38 MAPK. It also inhibits BACE-1 expression. These combined effects reduce Aβ accumulation, alleviate endoplasmic reticulum stress, attenuate neuroinflammation and neuronal apoptosis, and ultimately delay Alzheimer’s disease (AD) progression (Kang et al., 2013). Aβ deposition activates the MAPK cascade, including JNK, ERK, and p38, consequently inducing NF-κB activation, leading to glutamate excitability toxicity, synaptic plasticity disruption, proinflammatory cytokine production, and neuronal apoptosis, manifesting as cognitive dysfunction such as spatial learning and memory impairments in a rat model of AD. Treadmill exercise training inactivates MAPK and NF-κB signaling pathways, inhibits the production of proinflammatory cytokines and hippocampal apoptosis, and reduces cognitive impairment, such as spatial learning and memory (Kim et al., 2021). The MAPK signaling cascade is a complex process. At the same time, existing studies only describe the changes in the relevant signals *in vivo*; the specific process of how exercise training affects AD progression by inhibiting the MAPK cascade needs further study.

### 2.5 JAK/ STAT pathway

The Janus kinase (JAK)/ signal transducer and activator of transcription (STAT) pathway involves various pathophysiological processes in the central nervous system (mainly the cortex, hippocampus, and cerebellum). These include neurogenesis, glial cell generation and differentiation, synaptic plasticity, abnormal protein metabolism, mitochondrial function, inflammatory responses, and oxidative stress (Sarapultsev et al., 2023; Shahni et al., 2015). Bioinformatics analysis revealed that the AD group’s cytokine receptor interactions and JAK–STAT signaling pathways were functionally enriched (Tian et al., 2022). The JAK signal transducer and activator of transcription (JAK/STAT) pathway comprise transmembrane receptors, receptor-associated cytoplasmic tyrosine kinases (JAKs, including JAK1, JAK2, JAK3, and tyrosine kinase 2), and signal transducers and activators of transcription (STAT1, STAT2, STAT3, STAT4, STAT5A, STAT5B, and STAT6) (Leonard and O’Shea, 1998). Various cytokines, including interferon, interleukin (IL), and growth factors, all function in JAK–STAT signaling (O’Shea et al., 2002). Receptors activate JAKs by binding to extracellular ligands, which recruit, phosphorylate, and dimerize STAT, subsequently entering the nucleus to regulate the transcription of specific genes (Xue et al., 2023).

*In vivo*, the intraventricular administration of Aβ42 down-regulated p-STAT3, and p-STAT3 decreased in an age-dependent manner. At the same time, passive immunization with anti-Aβ42 antibody inversely restored the hippocampal p-STAT3 levels in Tg2576 mice, in parallel with the reduction of brain Aβ42 load and the recovery of Aβ42-induced memory impairment. Aβ42 consistently regulates p-STAT3 levels *in vitro*. Inhibition of JAK2/STAT3 axis not only leads to the loss of spatial working memory by down-regulating the acetylcholine-producing enzyme choline acetyltransferase, but also desensitizing the M1-type muscarinic acetylcholine receptor (Chiba et al., 2009). It was also found that Aβ42 inhibited the activation of JAK2 and STAT5 in the hippocampus of rabbits, thereby reducing the nuclear translocation of STAT5 and attenuating JAK2/STAT5 signaling. In contrast, leptin treatment significantly increased JAK2/STAT5 activation while also reversing the effect of Aβ42 on JAK2/STAT5 signaling (Marwarha et al., 2011). We also found that the phosphorylation of JAK2, STAT1, and STAT3 was significantly upregulated in the cerebral cortex and hippocampus of APP/PS1 transgenic mice, indicating the activation of the JAK/STAT signaling pathway. TREM2 overexpression attenuates neuroinflammatory responses by inhibiting the JAK2-STAT1/STAT3 signaling pathway (Ruganzu et al., 2021).

Cytokines, such as interferons, interleukins, and growth factors, and their receptors are all primary activators of JAK, while IL-6 is a potent activator of the JAK/STAT signaling pathway (Ding et al., 2022). Proinflammatory cytokines such as IL-6 enhance inflammation by mediating microglia activation and increasing Aβ, thus contributing to AD progression (McGeer and McGeer, 2015). IL-6 also mediates astrocyte production of hepcidin, a peptide hormone that functions as the critical regulator of iron homeostasis and is responsible for cellular iron uptake and release (Nemeth et al., 2004). Hepcidin is highly expressed in AD patients’ serum (Kweon et al., 2019; Delaby et al., 2025), but in the brain of AD, hepcidin expression is reduced and restricted to nerve cells, blood vessels, and damaged neurons (Raha et al., 2013). Hepcidin depletion in GFAP-positive cells during development leads to hippocampal atrophy and cognitive decline in mice (Bai et al., 2024). Studies have shown that the IL-6/STAT3 signaling pathway mediates brain neuroinflammation-induced iron dysregulation and hepcidin upregulation (You et al., 2017). Exercise training can reduce the expression of IL-6 in the brains of mice with AD (Hashiguchi et al., 2020) while significantly reducing STAT3/JAK1 levels (Belaya et al., 2021). Studies have found that hepcidin plays a dual role in brain iron load and inflammation, and inflammation may determine whether hepcidin has a harmful or beneficial effect on the brain. The effects of exercise training on hepcidin and inflammation need to be further studied. However, the relationship between AD and the JAK/STAT pathway requires further investigation. Simultaneously, the effect of exercise training on iron metabolism and the JAK/STAT pathway could play a crucial role in preventing and treating AD. However, the specific regulatory mechanism remains to be further explored.

### 2.6 PINK1-PARKIN pathway

In AD, Aβ deposition and accumulation of hyperphosphorylated tau protein induce excessive mitochondrial fragmentation and promote defective mitophagy. Mitophagy induced by various factors, such as stress and ubiquitin, also plays a role in the pathophysiology of AD. The Effective control of mitophagy could serve as a therapeutic target for AD (Pradeepkiran and Reddy, 2020; Sorrentino et al., 2017). Mutations in PINK1 and PARKIN mediate mitophagy, a prominent feature of AD (Moreira et al., 2010; Geisler et al., 2010). PINK1 and PARKIN signaling are ubiquitin-mediated mitophagy pathways driven by three major components: a mitochondrial damage sensor (PINK1), a signal amplifier (PARKIN), and a signal effector (ubiquitin chain) (Li et al., 2023). Following activation of the PINK1-PARKIN pathway, PINK1 phosphorylates ubiquitylated substrates on the outer mitochondrial membrane leads to recruitment of the E3 ligase PARKIN in coordination with its E2 ubiquitin-conjugating enzymes. PINK1 and PARKIN subsequently initiate a positive feedback loop to coat the phosphorylated ubiquitin chain in damaged mitochondria. This enables the selective autophagy of damaged mitochondria, selective removal of dysfunctional mitochondria, and the maintenance of intracellular mitochondrial homeostasis (Quinn et al., 2020). The PINK1/ PARKIN pathway can affect the metabolic process of phosphorylated tau and Aβ by regulating the activity of the mitophagy pathway (Zhou et al., 2023). Therefore, targeting the PINK1/PARKIN pathway provides a novel approach to preventing and treating AD. In the skeletal muscles, AMPK drives mitophagy through a PINK1-PARKIN independent mechanism by enhancing mitochondrial fission and autophagosomal engulfment (Seabright et al., 2020).

**Figure 5:**
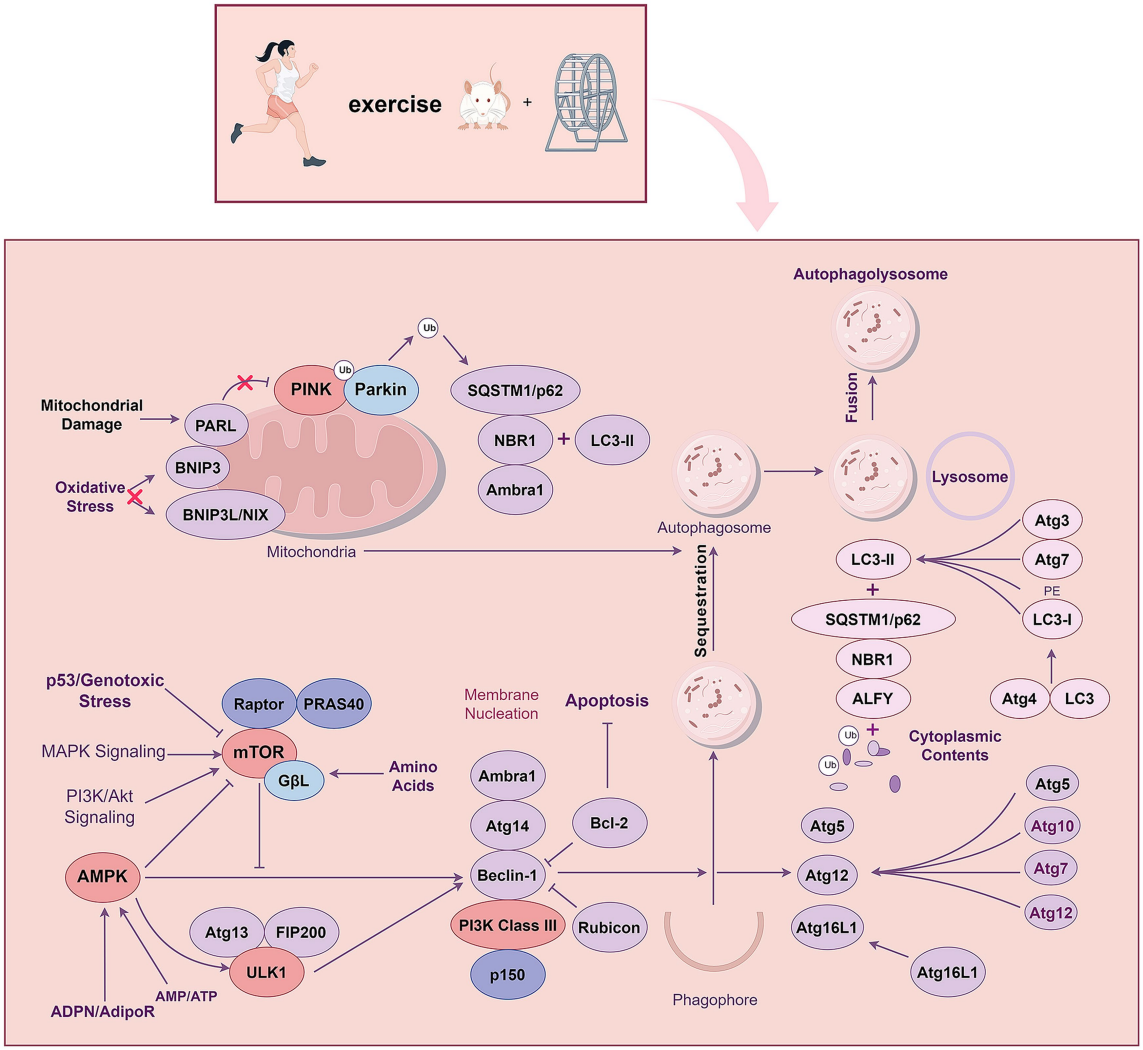
Exercise training regulates autophagy through PI3K/AKT, AMPK, MAPK and PINK1-Parkin signalling pathways.

Studies have further shown that the downstream target of AMPK, the SIRT1-FOXO1/3 axis, plays a vital role in exercise-enhanced PINK1/PARKIN pathway-mediated mitophagy in AD mice (Zhao et al., 2023), and the crosstalk between the PINK1/PARKIN pathway and other pathways needs to be further studied. Exercise training ameliorated mitochondrial dysfunction, reduced Aβ plaque area, and improved learning and memory ability by enhancing PINK1/PARKIN pathway-mediated mitophagy activity in the hippocampus of AD mice (Zhao et al., 2020). Using the lysosomal inhibitor chloroquine and the SIRT1 inhibitor EX527, researchers explored the underlying mechanism, finding that exercise training rescued PINK1/PARKIN pathway-mediated mitophagy by activating the SIRT1-FOXO1/3 axis in the hippocampus of AD mice. The lysosomal inhibitor chloroquine inhibits exercise-induced mitophagy (Zhao et al., 2021; Zhao et al., 2023). Activation of the PINK1/PARKIN pathway by exercise training may also be related to the activation of the AMPK pathway, while other pathways may affect the PINK1/PARKIN pathway-mediated mitophagy. In conclusion, activating the PINK1/PARKIN pathway-mediated mitophagy by exercise training is an alternative pathway for preventing and treating AD (Figure 5).

### 2.7 NF-κB pathway

The role of the NF-κB pathway in AD has attracted more and more attention. The NF-κB family is a family of transcription factors that includes five members: p65 (RelA), RelB, c-Rel, p105/p50, and p100/p52. The NF-κB pathway can be divided into classical and non-classical pathways with distinct activation mechanisms (Yu et al., 2020). The classical NF-κB pathway is closely related to cellular functions, such as inflammation, immune response, cell proliferation, differentiation, and survival (Hoesel and Schmid, 2013). In the pathophysiological process of AD, oxidative stress activates and increases the activation of microglia and astrocytes, thereby activating NF-κB, which is closely related to β-secretase activity and tau protein metabolism in the brain of AD patients.

Many researchers have elaborated on this in detail (Sun et al., 2022). Chronic systemic inflammatory response plays an essential role in the pathogenesis of AD, while effective measures to reduce this inflammatory response can prevent and treat AD. Exercise training can produce an adaptive response in the body related to the type of exercise training, intensity, and duration (Scheffer and Latini, 2020). Different exercise intensities can also produce anti-inflammatory and proinflammatory effects. Regular moderate-intensity exercise has beneficial effects (Pahlavani, 2023). Aβ deposition activates the MAPK cascade and the NF-κB pathway, disrupting synaptic plasticity, proinflammatory cytokine production, and neuronal apoptosis. Exercise training inactivates both the MAPK and NF-κB signaling pathways, inhibiting the production of proinflammatory cytokines and hippocampal apoptosis and alleviating cognitive dysfunction, such as spatial learning and memory (Kim et al., 2021).

Another study also found that exercise training could regulate MAPK-dependent signaling, leading to changes in nuclear NF-κB activity, thereby attenuating the activation of microglia and astrocytes, inhibiting the production of proinflammatory mediators and inflammatory factors, and inhibiting tau protein hyperphosphorylation (Leem et al., 2011).

The nucleotide-binding domain, leucine-rich repeat, and pyrin domai-containing protein 3 (NLRP3) inflammasome has been widely studied. Classical NF-κB induces the expression of NLRP3 in response to various stimuli. NLRP3 assembles the NLRP3 inflammasome, which clears pro-IL-1β to produce active cytokine IL-1β. The dysregulation of inflammasomes can lead to multiple autoinflammatory and autoimmune diseases. Exercise training can restore glucose hypometabolism-related memory impairment and tau hyperphosphorylation in diabetic rats by inhibiting the NF-κB/NLRP3 inflammatory pathway and stimulating the PI3K/Akt insulin pathway (Wang et al., 2019). The NF-κB pathway can also regulate inflammatory response by interacting with other signaling pathways (Wnt/β-catenin pathway, PI3K/Akt pathway, etc.). The relationship between the crosstalk of NF-κB pathway and different pathways and exercise training requires further investigation. Chronic neuroinflammation is a core mechanism underlying the development of AD. Exercise training exerts anti-inflammatory and neuroprotective effects through the NF-κB signaling pathway, which can be used as a critical target to slow AD progression.

### 2.8 TREM2 pathway

TREM2 is involved in multiple processes of AD pathophysiology, including amyloid and tau pathology, inflammatory responses, and microglial function (Haure-Mirande et al., 2022). TREM2, expressed in myeloid cells, is a transmembrane receptor of the immunoglobulin superfamily, comprising a V-type immunoglobulin domain, a short extracellular domain, a transmembrane helix, and a short cytoplasmic tail that does not contain any signal transduction or transport motifs (Deczkowska et al., 2020). TREM2, produced by microglia in the brain and expressed at a relatively high concentration in the hippocampus, spinal cord, and white matter, primarily regulates various microglial functions, including inflammatory cytokine production, microglial activation, and survival (Fujikawa and Tsuda, 2023). TREM2 directly interacts and binds with Aβ oligomers in the AD brain, activating TREM2 signaling (Qin et al., 2021). Among the identified susceptibility genes for AD, apolipoprotein E (APOE) and its allele subtype APOE4 have the greatest risk (Wightman et al., 2021). APOE accumulation occurs in the endolysosomal system of microglia (Kaji et al., 2024), and it can bind to TREM2 to promote microglia uptake of Aβ (Yeh et al., 2016) and antibody response (van Olst et al., 2025). Therapeutic strategies targeting TREM2 receptors are being extensively investigated, aiming to activate the receptor and stimulate microglial phagocytosis and clearance of Aβ deposits. Monoclonal antibodies against TREM2 agonists (AL002) is also being studied in Phase 2 efficacy and safety trial in patients with early AD (INVOKE-2; NCT04592874). Studies have examined the effects of four months of moderate-to-vigorous physical exercise on different markers of inflammatory processes in the cerebrospinal fluid and plasma of patients with AD, with some finding that exercise increases sTREM2 levels in the cerebrospinal fluid of patients with AD (Jensen et al., 2019).

Studies have shown that TREM2 was upregulated in AD mice’s hippocampus after exercise training (Wang et al., 2023b). Furthermore, exercise can inhibit the shedding of TREM2 and maintain TREM2 protein levels in a manner potentially related to the regulation of glucose metabolism in the hippocampal brain and microglia. At the same time, the morphological plasticity of hippocampal microglia is also regulated by exercise training in AD mice (Zhang et al., 2022b). In animal models of AD, Aβ-induced recognition memory impairment was associated with neuroinflammation induced by hippocampal microglia activation, while exercise training could switch microglia from M1 to M2 phenotype. It also reduces neuroinflammation in AD via the HSP60/TREM2/DAP12 pathway (Zhang et al., 2022a). Aβ-induced AD rats show recognition memory impairment. Treadmill exercise preconditioning can regulate the expression of TREM2 in the hippocampus of rats to prevent the decline of recognition memory in AD rats. Treadmill exercise pretreatment also rescued the reduced dendritic complexity, spine density, synaptic protein expression, synaptic ultrastructure and neurotransmitter expression in the hippocampus of AD model (Zhang et al., 2025). The TREM2 pathway may be an essential regulator of the phenotypic transformation of microglia during the pathophysiological process of AD and a potential therapeutic target for AD.

## 3 Summary and future prospects

Signaling pathways regulate various cell cycle processes through a series of enzymatic reactions that transmit extracellular molecular signals to cells to exert numerous effects, playing an essential role in the pathophysiology of AD. Moderate intensity and long-term continuous exercise training can effectively reduce the prevalence of AD and improve its symptoms. Several studies have explored the relationship between exercise training and AD. This review summarizes the mechanisms and potential of exercise training to exert beneficial effects on AD from the perspective of different signaling pathways. Exercise training regulates multiple signaling pathways related to AD pathophysiology, including the PI3K/Akt, Wnt/β-catenin, AMPK-related, MAPK, NF-κB, PINK1-PARKIN, JAK/STAT, and TREM2 signaling pathways (Figure 1). Interconnected signaling pathways crosstalk via distinct targets to combat AD pathogenesis: inhibition of Aβ plaques, reduction of hyperphosphorylated tau and apoptosis, mitigation of neuroinflammation, and restoration of autophagy. These synergistic effects ultimately ameliorate cognitive decline in AD.

Many of the studies discussed in this article have primarily explored the effects of exercise training on different signaling pathways in the pathophysiological process of AD in animal models. However, a significant gap still needs to be seen between animal studies and studies on patients with AD regarding relevance and sample size. In the future, more human studies are required to provide a firmer biological basis to support the benefits of exercise training. Given the complexity of the pathophysiological processes of AD, exercise training with multitarget therapeutic potential has become an effective treatment strategy. A number of studies have shown that moderate-intensity aerobic exercise is an effective exercise method for the treatment of AD (Jia et al., 2025; Jiang et al., 2025), but the timing and intensity of exercise intervention in AD treatment need further research and clarity; however, it is undoubtedly an effective treatment. Different signaling pathways, such as TREM2, are closely related to delays in AD progression. However, studies on preventing and treating AD through distinct signaling pathways are relatively scarce; this is a promising direction for future research. However, owing to the complexity of the AD pathophysiology, a single kinase modulator may not exert a therapeutic effect, and a multi-pathway comprehensive treatment method needs to be further explored.

In summary, this review of the signaling pathways underlying the beneficial effects of exercise training as an intervention for AD will help to explore the optimal exercise prescription for the prevention and treatment of AD and provide a reference for the future development of novel, effective prevention and treatment targets for AD, thereby developing promising personalized, combined intervention strategies, including effective therapeutic drugs, functional foods, and exercise mimetics.

## Glossary

ACSS2: AMPK-mediated acetyl-CoA synthetase 2
AD: Alzheimer’s disease
AdipoR1: Adiponectin receptor 1
ADPN: Adiponectin
Akt: Protein kinase B
AMPK: Adenosine 5′-monophosphate-activated protein kinase
APP: Amyloid precursor protein
Aβ: Amyloid peptides-β
BACE: β-Secretase
Bax: Bcl-2-associated X protein
Bcl-2: B-cell lymphoma-2
BDNF: Brain derived neurotrophic factor
CHIP: Carboxyl terminus of HSP70 interacting protein
Dkk: Dickkopf
ERK: Extracellular signal-regulated kinase
FNDC5: Fibronectin type III domain containing 5
FOXO: Forkhead box O
GSK-3β: Glycogen synthase kinase-3β
HSF1: Heat shock transcription factor 1
HSP70: 70-kDa heat shock protein
IDO: Indoleamine-2,3-dioxygenase
IGF: Insulin like growth factor
IL: Interleukin
JAK: Janus kinase
JNK: c-Jun N-terminal kinase
LRP6: Low-density lipoprotein receptor-related protein 6
MAPK: Mitogen activated protein kinase
mRNA: Messenger ribonucleic acid
mTOR: Mammalian/mechanistic target of rapamycin
mTORC: mTOR complex
NF-κB: Nuclear factor kappa B
NLRP3: NBD-, LRR- and PYD-containing 3
NSE: Neuron-specific enolase
PARKIN: Parkin RBR E3 ubiquitin-protein ligase
PGC-1α: Peroxisome proliferator-activated receptor γ coactivator-1α
PI3K: Phosphatidylinositol 3-kinase
PINK1: PTEN-induced kinase 1
PLC: Phospholipase C
PP2A: Protein phosphatase 2A
PS1: Presenilin-1
P-tau: Hyperphosphorylated tau protein
ROS: Reactive oxygen species
SD: Sprague Dawley
SIRT1: Sirtuin1
STAT: Signal transducer and activator of transcription
SYN: Synaptophysin
TFEB: Transcription factor EB
TREM2: Triggering receptor expressed on myeloid cells 2
UCHL-1: Ubiquitin carboxyl-terminal hydrolase L1
UPR: Unfolded protein response
UPS: Ubiquitinsingle bondproteasome system
βCTF: β-C-terminal fragment
